# Supplementary material for: Analysis of Intestinal Mucosa Integrity and GLP-2 Gene Functions upon Porcine Epidemic Diarrhea Virus Infection in Pigs
Source: Animals (Basel). 2021 Mar 1;11(3):644. doi: 10.3390/ani11030644 (PMC8000733; doi:10.3390/ani11030644)
Supplement: Supplementary file 1 [file animals-11-00644-s001.pdf]

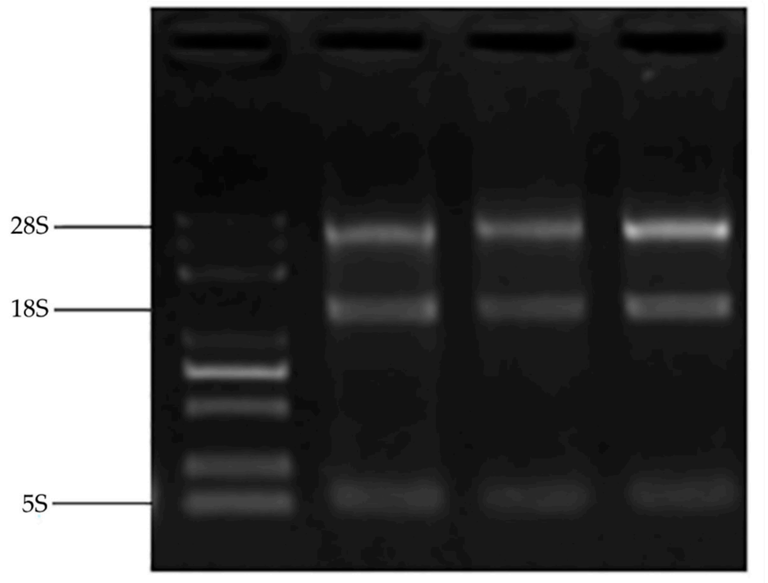

Figure S1. Gel electrophoresis of tissue total RNA

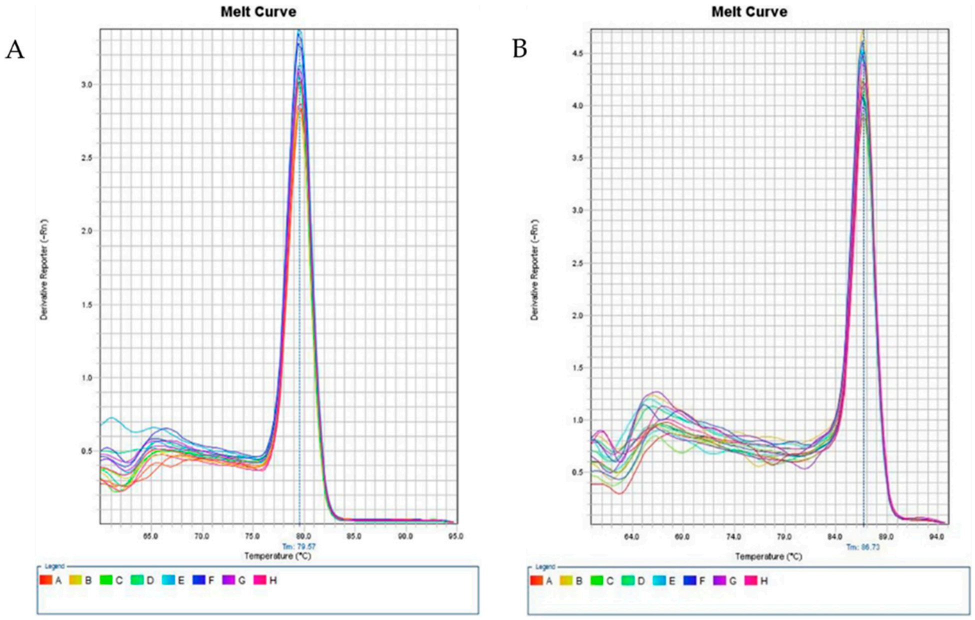

Figure S2. qPCR amplification curves**.** A and B represent *GLP-2* and PEDV M genes, respectively

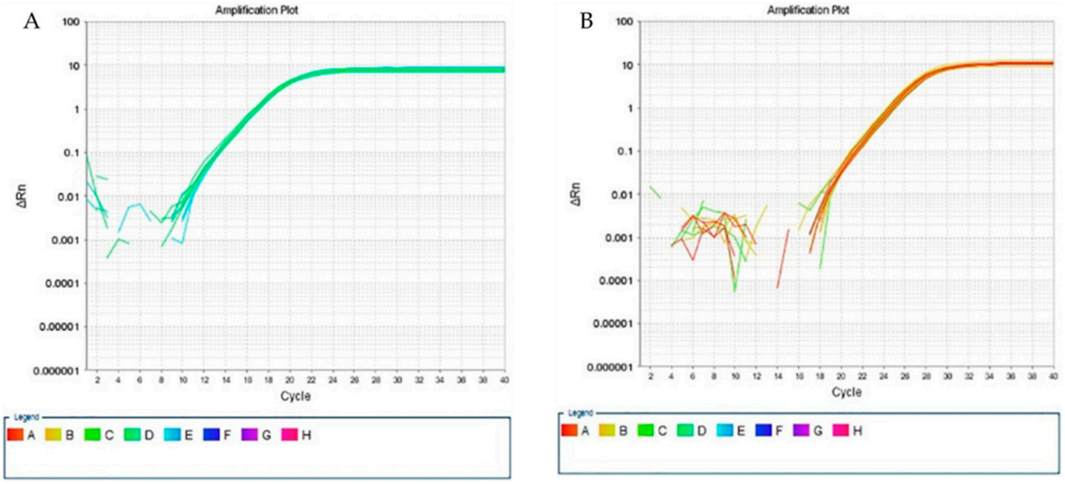

Figure S3. qPCR melting curves. A and B represent *GLP-2* and PEDV M genes, respectively
